# Behavior of protein-polysaccharide conjugate-stabilized food emulsions under various destabilization conditions

**DOI:** 10.1016/j.fochx.2023.100725

**Published:** 2023-05-29

**Authors:** Majid Nooshkam, Mehdi Varidi, Zahra Zareie, Fatemeh Alkobeisi

**Affiliations:** a Department of Food Science and Technology, Faculty of Agriculture, Ferdowsi University of Mashhad (FUM), Mashhad, Iran; b Department of Food Science and Technology, Faculty of Food Science and Technology, Gorgan University of Agricultural Sciences and Natural Resources, Gorgan, Iran

**Keywords:** Maillard reaction, Protein-polysaccharide conjugates, Interfacial properties, Emulsions, Steric repulsion, Interfacial antioxidant

## Abstract

The sensitivity of protein-stabilized emulsions to flocculation, coalescence, and phase separation under destabilization conditions (i.e., heating, aging, pH, ionic strength, and freeze-thawing) may limit the widespread use of proteins as effective emulsifiers. Therefore, there is a great interest in modulating and improving the technological functionality of food proteins by conjugating them with polysaccharides, through the Maillard reaction. The present review article highlights the current approaches of protein-polysaccharide conjugate formation, their interfacial properties, and the behavior of protein-polysaccharide conjugate stabilized emulsions under various destabilization conditions, including long-term storage, heating and freeze-thawing treatments, acidic conditions, high ionic strength, and oxidation. Protein-polysaccharide conjugates are capable of forming a thick and cohesive macromolecular layer around oil droplets in food emulsions and stabilizing them against flocculation and coalescence under unfavorable conditions, through steric and electrostatic repulsion. The protein-polysaccharide conjugates could be therefore industrially used to design emulsion-based functional foods with high physicochemical stability.

## Highlights

• Conjugates form thick interfacial layer and provide high physicochemical stability.
• Conjugate increases mechanical strength and viscoelasticity of interfacial film.
• Conjugates stabilize emulsions by strong and long-range steric interactions.
• Conjugate-stabilized emulsions increase oxidative stability of bioactive compounds.

## 1 Introduction

Emulsions are hugely applied in the food and medical industries to encapsulate and deliver biologically active compounds, alter optical properties, and modify rheological and organoleptic attributes. Traditionally, conventional emulsions (oil-in-water; O/W or water-in-oil; W/O) are used in industrial applications and their small spherical droplets (100 nm to 100 μm) are coated by a single layer of emulsifiers, stabilizing them against aggregation. However, the conventional emulsions are thermodynamically unstable and tend to break down over time due to flocculation, coalescence, Ostwald ripening, gravitational separation, and phase inversion (McClements, 2012, McClements and Li, 2010, Nooshkam and Varidi, 2020a).

O/W emulsions have been extensively applied in food, cosmetic, and pharmaceutical industries. In this way, hydrophobic nutrients and drugs can be encapsulated in oil droplets of O/W emulsions to improve their solubility and stability in aqueous-based food products. Food proteins contain hydrophobic and hydrophilic amino acids along their polypeptide chains, and they are therefore surface-active molecules with the ability to rapidly adsorb to the oil–water interfaces and create thin electrically charged interfacial layers (Ozturk & McClements, 2016). Although the functional and interfacial properties of proteins in food emulsions have been traditionally investigated in relatively simple but well-defined model systems, the protein-stabilized emulsions tend to industrially experience a wide variety of conditions and environmentally stresses (e.g., pH, ionic strength, biopolymers, and surfactants, and aging, thermal, freezing, chilling, and drying treatments) from production to utilization stages (McClements, 2004). Indeed, protein-coated oil droplets could undergo creaming, coalescence, Ostwald ripening, and flocculation destabilization mechanisms, of which the droplet flocculation is the most subtle and complicated one to control. This is due to the fact that the flocculation state could be sensitively affected by various factors, and it can also have a direct influence on the emulsion structure and other instability mechanisms, particularly creaming and coalescence (Dickinson, 2010). The sensitivity of protein-stabilized emulsions to flocculation, coalescence, and phase separation after heat treatment and changes in pH and ionic strength of the system, could therefore limit the common use of proteins as emulsifying agents (Liu et al., 2016, Wang et al., 2016). Therefore, there is a great interest in modulating and ameliorating the technological functionality of food proteins by conjugating them with food-grade polysaccharides, through the Maillard reaction (Einhorn-Stoll et al., 2005, Nooshkam and Varidi, 2020b, O’Regan and Mulvihill, 2009, Xi et al., 2020).

The protein-polysaccharide conjugates have an enhanced emulsifying property and emulsion stability towards flocculation, coalescence, and creaming, mainly due to the steric and electrostatic repulsive interactions between oil droplets of the emulsions (Hu et al., 2022, Jiang et al., 2023, Kim and Shin, 2021). The polysaccharide moiety in the protein-polysaccharide conjugates provide strong steric and sometimes electrostatic repulsions, and the protein part can be attached to hydrophobic surfaces (i.e., oil droplets) (Jiang et al., 2023, Nooshkam and Varidi, 2020a, Yao et al., 2022, Zhang et al., 2022). These characteristics make them excellent polymers for the emulsification and stabilization of unstable bioactive components, as the adsorbed hydrocolloids act as a thickening, gelling, and structuring agent in O/W emulsions (Nooshkam, Varidi, & Verma, 2020). The protein-polysaccharide conjugates could be therefore considered as a promising structural design principle to fabricate emulsions with novel functional properties. For example, the soy hull hemicelluloses-soy protein isolate (SHH-SPI) conjugate showed a significant improvement in emulsification ability compared to SHH and SPI alone in maintaining the physical stability of the O/W emulsion for a long-time during heat treatment (Wang, Wu, & Liu, 2017). Additionally, compared to using protein as a stabilizer, emulsions containing bioactive compounds stabilized by protein-polysaccharide conjugates have improved pH and thermal stability (Chen et al., 2022, Nooshkam and Varidi, 2021a).

This work differs significantly from other existing review articles as it sheds light on the stability of protein-polysaccharide conjugate-coated oil droplets in the food emulsion under various destabilizing conditions (e.g., storage, heating, freeze–thaw, pH and ionic strength changes, dilution, and oxidation) in one spot. This fact is not observed in other review articles like those provided by Kan et al., 2021, Feng et al., 2022, and Sun et al. (2022). This review paper, therefore, highlights the behavior of protein-polysaccharide conjugate stabilized emulsions under various destabilization conditions.

## 2 Protein-polysaccharide conjugates

The Maillard reaction (also known as the Maillard glycation, Maillard conjugation, and non-enzymatic browning reaction) includes early, intermediate, and final stages, which their mechanisms and characteristics can be seen in previous reviews (Nooshkam et al., 2019, Nooshkam et al., 2020). Protein conjugation is commonly performed at the initial stage of the Maillard reaction, and the native protein not only preserves its technological function but also has improved functional features with the aid of polysaccharides. It is also worth mentioning that the relatively low reducing potential and high molecular steric hindrance of polysaccharides compared with mono- and disaccharides prevent the advanced Maillard reaction from occurring and inhibit the degradation of protein-polysaccharide conjugate during glycation reaction. In addition, natural and safe substrates are used to form protein-polysaccharide conjugates, and no hazardous and toxic advanced reaction products are obtained under controlled reaction conditions; thus, protein-polysaccharide conjugates could be applied as effective ingredients to produce novel foods with safe and healthy characteristics (de Oliveira et al., 2016, Qi et al., 2010, Zhang et al., 2019).

### 2.1 Preparation procedures

Protein-polysaccharide conjugates are traditionally prepared by “dry-heating” and “wet-heating” methods. However, other methods such as microwave-, ultrasound-, pulsed electric field-, irradiation-, and cold atmospheric plasma-assisted methods, which are generally based on the wet-heating mode of the Maillard reaction, are also currently used to prepare protein-polysaccharide conjugates with improved emulsifying properties (Table 1).

**Table 1:** Summarization of protein-polysaccharide conjugates with improved emulsifying properties obtained through the different preparation methods.

Protein type	Polysaccharide type	Preparation/heating method	Emulsion characteristics	Reference
Ovalbumin	Dextran	Dry heating	• The ovalbumin–dextran conjugate retained excellent emulsifying properties even at pH 3 and was further enhanced at pH 10.	(Kato, Sasaki, Furuta, & Kobayashi, 1990)
Sodium caseinate	Pectin	Dry heating	• Despite elevated temperature, high salt buffers, and low pH, the conjugate had better emulsifying properties than sodium caseinate or pectin alone or the commercial emulsifiers gum Arabic and glycerol monostearate. • The conjugate produced emulsions with smaller droplets than sodium caseinate and pectin emulsions.	(Al-Hakkak & Kavale, 2002)
Whey protein isolate (WPI)	Dextran	Dry heating	• The conjugate stabilized emulsion had much better storage, pH, and electrolyte stabilities than the WPI alone or gum Arabic.	(Akhtar & Dickinson, 2003)
WPI/sodium caseinate	Pectin	Dry heating	• WPI-pectin conjugate had the best emulsifying properties. • In combination with any pectin, sodium caseinate did not produce glycoproteins with improved emulsifying properties.	(Einhorn-Stoll, Ulbrich, Sever, & Kunzek, 2005)
Soy protein isolate	Dextran	Dry heating	• The conjugate inhibited the droplet-protein aggregate interaction and floc formation in the emulsions heated at 100 °C, in comparison to protein and protein-polysaccharide mixture stabilized emulsions.	(Diftis & Kiosseoglou; 2006)
Sodium caseinate	Maltodextrin	Dry heating	• The conjugate stabilized emulsions and liqueurs showed improved storage stability when compared to sodium caseinate stabilized emulsions and liqueurs under accelerated shelf-life testing conditions (45 °C for 20 and 90 days).	(O’Regan & Mulvihill, 2009)
WPI/sodium caseinate	Dextran/pectin	Dry heating	• The emulsifying activity of milk proteins increased upon *Maillard* modification with pectin (up to + 1242%) and dextran (up to + 513%).	(Hiller & Lorenzen, 2010)
WPI/β-lactoglobulin	Corn fiber gum	Dry heating	• At room temperature, the conjugates stabilized emulsions better than corn fiber gum or protein alone based on measurements of turbidity, particle size distribution, and average particle size.	(Yadav, Parris, Johnston, Onwulata, & Hicks, 2010)
Wheat protein isolate	Dextran	Dry heating	• The conjugate formed a thicker interfacial layer and provided enhanced steric stabilization of the emulsions at acidic pH (∼pH 4).	(Wong, Day, & Augustin, 2011)
β-lactoglobulin	Gum Acacia Seyal	Dry heating	• β-lactoglobulin conjugation significantly improved the stability and resistance of gum Acacia Seyal stabilized emulsions to low pH and high salinity conditions.	(Bi, Yang, Fang, Nishinari, & Phillips, 2017)
Sodium caseinate	Locust bean gum	Dry heating	• The conjugate provided high stability in O/W emulsions at pHs 3.5 and 7 probably due to the formation of a thicker interfacial layer, which provided an increase in steric and electrostatic repulsion between the droplets.	(Barbosa, Ushikubo, de Figueiredo Furtado, & Cunha, 2019)
Bovine serum albumin	Fucoidan	Dry heating	• The conjugates improved the thermal stability of the emulsion than intact protein and protein-polysaccharide mixtures.	(Kim & Shin, 2021)
WPI	Gum Acacia	Dry heating	• The stability of emulsions during freeze–thaw processing, high ionic strength conditions, heat treatment and pH conditions close to the WPI isoelectric point were improved by glycation. • The chemical stability of β-carotene in the emulsion was also improved by the WPI-gum Acacia conjugate. • The stabilized emulsion of WPI-gum Acacia conjugate increases the bioavailability of β-carotene by providing better stability to droplet aggregation during simulated *in vitro* gastrointestinal digestion.	(Chen et al., 2022)
Egg white proteins	Dextran	Dry heating	• The emulsions prepared using the conjugate at a 1:1 mass ratio showed better physical stability under neutral conditions than those prepared with the protein alone. • They also showed strong tolerance to changes in environmental conditions, including pH, salinity, and temperature.	(Yao et al., 2022)
Soy protein isolate	Dextran	Wet heating	• The conjugate had higher emulsifying activity at high temperature, high ionic strength, and pHs 3, 7, and 10, compared to the protein alone.	(Qi, Liao, Yin, Zhu, & Yang, 2010)
Wheat germ protein	Dextran	Wet heating	• The conjugate had significantly higher emulsifying activity and emulsion stability that the protein.	(Niu, Jiang, Pan, & Zhai, 2011)
WPI	Dextran	Wet heating	• The WPI-dextran conjugate stabilized emulsions had remarkably lower droplet size and higher storage stability compared to those stabilized by WPI.	(Zhu, Damodaran, & Lucey, 2010)
WPI	Gellan gum	Wet heating	• The conjugated WPI had an improved interfacial activity and emulsifying activity.	(Nooshkam & Varidi, 2020b)
WPI	Gellan gum	Wet heating	• The emulsion droplets coated by the conjugate were generally more stable to aging, pH changes, ionic strength, freeze–thaw cycles, and thermal treatment than the WPI stabilized emulsion.	(Nooshkam & Varidi, 2021a)
Pea protein isolate	Maltodextrin	Wet heating	• The emulsifying properties of pea protein isolate were greatly improved after conjugation. • The conjugate-stabilized canola oil-in-water emulsion showed the highest physical stability when exposed to 25–60 °C.	(Zhang et al., 2022)
Fish gelatin	Xanthan gum/ guar gum/ konjac glucomannan	Wet heating	• The conjugate showed better creaming stabilization ability than pure fish gelatin and polysaccharide. • The creaming stability of conjugated stabilizing emulsions depends on the Maillard reaction time and the type of polysaccharide.	(Hu et al., 2022)
Whey protein concentrate	Carboxymethyl cellulose	Wet heating	• Protein emulsification performance was improved by glycosylation and was confirmed by emulsification activity (4.48% increase), particle size and zeta potential.	(Jiang et al., 2023)
β-conglycinin	Dextran	High pressure	• The emulsification activity of β-conglycinin was improved by dextran conjugation under high pressure treatment.	(Xu et al., 2010)
WPI	Gum Acacia	Ultrasound	• The ultrasound-derived conjugates had manifestly higher emulsifying activity and emulsion stability compared to those obtained from classical heating.	(Chen et al., 2019)
Pea protein isolate	Inulin	Ultrasound	• The conjugate-stabilized emulsion exhibited shear-thinning behavior and had a higher viscosity than the protein-stabilized emulsion. • They also had a higher surface charge and formed a thicker interfacial layer, and significantly improved the physical and oxidative stability of the emulsion.	(Jiang et al., 2022)
WPI	Dextran	Pulsed electric field	• WPI–dextran conjugates had better emulsifying capabilities for O/W emulsion at pH 7.0 than initial WPI.	(Sun, Yu, Zeng, Yang, & Jia, 2011)
Bovine serum albumin	Starch	Pulsed electric field	• The emulsions stabilized by the conjugates had smaller droplet sizes and higher adsorbed protein with improved emulsion stability. • The emulsions stabilized by the conjugates showed better stability at pH = 4.6 and against different ionic strengths (150–300 mM NaCl).	(Taha et al., 2022)

#### 2.1.1 Dry-heating mode

The protein conjugation *via* the dry heating mode was first introduced between ovalbumin and dextran in the 1990s (Kato, Sasaki, Furuta, & Kobayashi, 1990). After that, the dry-heating version of the Maillard reaction has been usually adopted and regarded as the major method to prepare protein-polysaccharide conjugates under controlled relative humidity (RH) and temperature. In this method, polysaccharide and protein aqueous solutions (at the desired ratio, depending on the reactant types) are lyophilized and then powdered to perform the conjugation under controlled conditions, including temperature (40–80 °C), RH (usually 65% or 79%), and time (several hours to weeks), followed by cooling to room temperature to cease the conjugation reaction (de Oliveira et al., 2016).

The dry heating mode is extensively applied as a straightforward procedure and the final product exhibits more feasibility for handling and storage along with a long shelf-life (Karbasi & Madadlou, 2018). However, (1) the conjugation time takes several days to weeks, (2) the reaction extent is uncontrollable (possibly resulting in extensive browning development and the final product is usually a mixture of intermediate and/ or advanced Maillard reaction products), (3) it requires a costly freeze/spray-drying step and controlled humidity and temperature during the reaction, (4) uneven contact between reactants could limit the reaction extent, and (5) rigid or compact proteins could lead to inefficient conjugation (Perusko, Al-Hanish, Cirkovic Velickovic, & Stanic-Vucinic, 2015). From an industrial viewpoint, the dry-heating processing is costly and not attractive and/ or applicable for mass production, which largely hampers commercial applications of protein-polysaccharide conjugates as potential food ingredients (Wang and Xiong, 2016, Zhuo et al., 2013).

#### 2.1.2 Wet-heating mode

The wet-heating method has been therefore adopted to address these problems and prepare protein-polysaccharide conjugates with improved functional properties. This mode of the Maillard conjugation largely shortens the incubation time to only several hours at high temperatures, which can limit the conjugation reaction to the initial stage of Schiff base formation and provide better control of brown color development; thus, this mode of heating would be more viable for the food industry (Chen et al., 2017, Nooshkam and Madadlou, 2016a, Nooshkam and Madadlou, 2016b, Xu et al., 2018). However, the protein denaturation and polymerization at high temperatures and/or long treatment time is the main drawback of the wet-heating method, which could affect the functional properties of the protein-polysaccharide conjugates (Chen et al., 2019). The protein denaturation/polymerization extent could be however reduced by the macromolecular crowding effect, which could preserve the native structure of the proteins during heating of protein-polysaccharide aqueous solution (O’Mahony, Drapala, Mulcahy, & Mulvihill, 2019). It could be necessary to note that the Maillard reaction rate between polysaccharides and proteins in an aqueous solution is low, and it is, therefore, feasible to cease the conjugation reaction at the initial stage by rapidly lowering the temperature to approximately 0 °C. It is therefore expected that the steric factors would stabilize the Schiff base formed between protein’s amino group and bulky polysaccharide’s reducing-end carbonyl group (Zhu et al., 2008, Zhu et al., 2010).

#### 2.1.3 Novel conjugation methods

The protein conjugation through the conventional methods either needs a high cost (due to the freeze-drying step) or results in low conjugation yields. In addition, the uncontrolled long dry/wet-heating periods could lead to the generation of undesired products, such as brown and hazardous compounds (Oliver, Melton, & Stanley, 2006). In this context, other conjugation approaches, such as irradiation, ultrasonication, high pressure, and pulse electric field, could effectively promote the protein conjugation reaction to address the limitations and disadvantages of the conventional procedures (Chen et al., 2019, Sun et al., 2011, Taha et al., 2022, Xu et al., 2010, Zhang et al., 2019).

### 2.2 Properties

Protein-polysaccharide Maillard-type conjugates usually have improved solubility, thermal stability, thickening ability, foaming capacity, emulsifying properties, antimicrobial effect, and antioxidant activity. For more information on the technological function and biological properties of protein-polysaccharide conjugates, readers can refer to recent publications by Nooshkam et al., 2019, Nooshkam et al., 2020, and Zhang et al. (2019). The emulsifying and interfacial properties of the protein-polysaccharide conjugates are comprehensively discussed in the following sections.

## 3 Protein-polysaccharide conjugate-stabilized emulsions

### 3.1 Structural organization

The protein structure usually undergoes some conformational changes during the conjugation reaction to adopt flexible structures with a higher rate of adsorption to the O/W interface compare with the native protein (Nooshkam et al., 2020). The Maillard reaction could disrupt some structural interactions in proteins (e.g., hydrophobic interactions, hydrogen bonds, electrostatic attraction, Van der Waals forces, and covalent bonds), enabling them to become more flexible (Li et al., 2019a). The flexibility and surface hydrophobicity are considered the most essential structural factors in increasing the interfacial properties of protein-polysaccharide conjugates (Li et al., 2019a, Li et al., 2019b, Nooshkam and Varidi, 2021b, O'Mahony et al., 2017). The flexible conjugates with higher surface hydrophobicity could therefore improve hydrophobic interactions between oil droplets and proteins, and in turn decrease interfacial tension more rapidly than the non-conjugated protein during emulsification (Nooshkam & Varidi, 2020b). The polysaccharide moiety renders strong steric and electrostatic repulsion in protein-polysaccharide conjugates, and the protein moiety can attach to hydrophobic surfaces (Nooshkam & Varidi, 2021b).

### 3.2 Stabilization mechanisms

#### 3.2.1 Steric and electrostatic repulsion

**Fig. 1:**
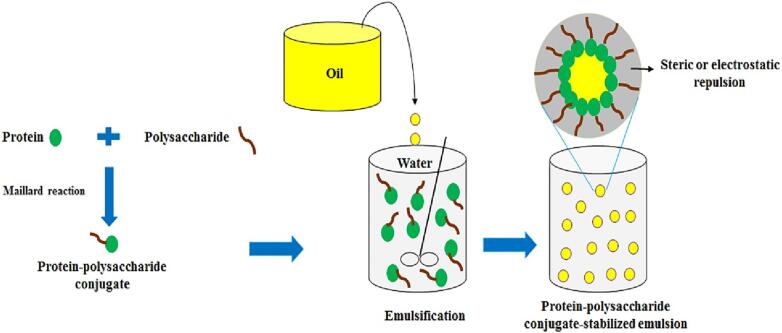
O/W emulsion droplets coated by native protein and protein-polysaccharide conjugates. Reproduced from ref. (Nooshkam & Varidi, 2020a) with permission from Elsevier Science Ltd. Copyright 2020.

The O/W emulsions stabilized by protein-polysaccharide conjugates consist of small oil droplets surrounded by a cohesive, thicker, and viscoelastic interfacial layer, which typically originates from protein-polysaccharide conjugates. This interfacial layer confers strong steric repulsive forces and sometimes electrostatic repulsion (in case of anionic polysaccharides) between oil droplets, thereby preventing their aggregation and coalescence under various destabilization conditions (Lavaei, Varidi, & Nooshkam, 2022) (Fig. 1). As a result of the Maillard reaction, the predominant stabilization mechanism changes from electrostatic repulsion in protein-coated droplets to strong steric hindrance in conjugate-stabilized pairs (Caballero & Davidov-Pardo, 2021). The high physiochemical stability of protein-polysaccharide conjugate stabilized emulsions has extensively been attributed to the strong steric (and sometimes electrostatic) repulsion between oil droplets provided by the conjugate (Chen et al., 2022, Caballero and Davidov-Pardo, 2021, Lavaei et al., 2022, Hu et al., 2022, Jiang et al., 2023, Kim and Shin, 2021).

#### 3.2.2 Thickening effect

The emulsion viscosity also plays an important role in maintaining emulsion stability, and the higher the molecular weight of the emulsifier, the higher the viscosity of the emulsion (Lin, Yu, Ai, Zhang, & Guo, 2020). In this case, the high molecular weight of the protein-polysaccharide conjugate may result in an emulsion with greater physical stability (Ai, Zhao, Guo, Chen, & Yu, 2022). According to Boostani and colleagues (2017), the covalent bonding between dextran and soy proteins could result in increases in viscosity, which would increase the stability of the emulsion (Boostani, Aminlari, Moosavi-nasab, Niakosari, & Mesbahi, 2017).

## 4 Physicochemical stability of protein-polysaccharide conjugate-stabilized emulsions

### 4.1 Long-term storage (aging)

Protein molecules could undergo considerable conformational (surface denaturation) and interaction changes after adsorption to oil–water interfaces as a consequence of changes in their molecular environment, to maximize the favorable interactions and minimize the unfavorable interactions in its new environment (McClements, 2004, Ozturk and McClements, 2016). The hydrophobic and sulfhydryl-containing amino acids could be unmasked by the surface denaturation and, in turn, trigger flocculation in protein-stabilized O/W emulsions *via* increased disulfide bond formation and hydrophobic attraction between proteins adsorbed onto different droplets when are in close proximity (e.g., pH ∼ pI or high ionic strength) (McClements et al., 1993, Nejadmansouri et al., 2016). This could affect the physicochemical properties and the appearance of protein-stabilized emulsion-based food products during long-term storage. Protein-polysaccharide conjugate is capable of significantly increasing the stability of O/W emulsions against storage-triggered droplet coagulation, by inducing strong electrostatic and steric repulsion between oil droplets. The higher storage stability of protein-polysaccharide conjugate-stabilized emulsions than those stabilized by native protein has been extensively reported in the literature (An et al., 2014, Seidi et al., 2023, Yadav et al., 2010, Yao et al., 2022).

**Fig. 2:**
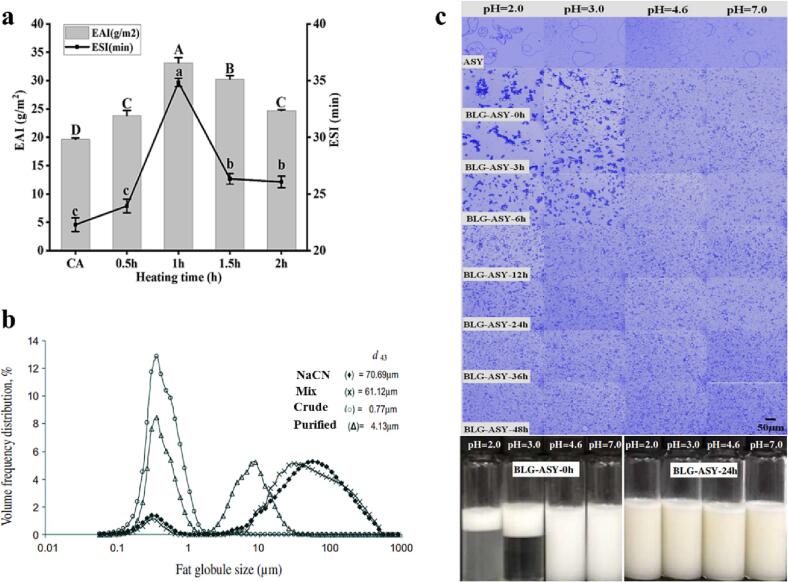
(a) Emulsifying activity index (EAI) and emulsion stability index (ESI) of casein (CA) and CA-arabinogalactan conjugates. Reproduced from ref. (Yang et al., 2023) with permission from Elsevier Science Ltd. Copyright 2023. (b) Fat globule size distribution and mean fat globule size (d_43_) of emulsions stabilized by NaCN, mixture, crude conjugate, and purified conjugate following storage at 45 °C for 20 days. Reproduced from ref. (O’Regan & Mulvihill, 2010) with permission from Elsevier Science Ltd. Copyright 2010. (c) pH-tolerance of the emulsions stabilized with pure ASY or BLG-ASY conjugates of different incubation times. Reproduced from ref. (Bi et al., 2017) with permission from Elsevier Science Ltd. Copyright 2017.

By conjugating egg white proteins to pectin, Al-Hakkak and Al-Hakkak (2010) reported that egg white protein-stabilized O/W emulsion was only stable for three weeks at room temperature and further storage time led to a visual phase separation in the system; whilst, the emulsion stability improved from 3 to 18 weeks when the protein-pectin conjugates were used as an effective emulsifier. This is mainly because protein-polysaccharide conjugates, with a new hydrophilic-lipophilic balance, can create a stabilizing macromolecular layer around the oil droplets and stabilize them against coalescence and flocculation during storage, through electrostatic and steric repulsive forces (Chen et al., 2020, Chen et al., 2019). However, longer conjugation times resulted in the conjugates with remarkably lower solubility and weaker physical structure and in turn with failed ability to produce a stable emulsion (Al-Hakkak & Al-Hakkak, 2010). This means that the Maillard reaction should be carefully controlled to fabricate protein-polysaccharide conjugates with improved emulsifying activity and emulsion stability. Similar results have been reported by Bi, Yang, Fang, Nishinari, and Phillips (2017), who worked on the storage stability of O/W emulsions stabilized by β-lactoglobulin (BLG) or BLG-gum *Acacia Seyal* (ASY) conjugates. It was consistently reported that the highest emulsifying activity/emulsion stability and degree of glycosylation were achieved when casein conjugates were formed by heating with arabinogalactan for 1 h (Fig. 2a). And the excessive heating reaction led to a significant decrease in the emulsion formation/stability, mainly due to the gradual increase in the surface hydrophobicity induced by the decrease in the degree of glycation and a greater denaturation of the protein (exposure of hydrophobic groups) (Yang et al., 2023).

The increased stability of food emulsions containing protein-polysaccharide conjugates could be also attributed to the improved viscosity of proteins after the conjugation reaction. In this context, viscosity can reduce the gravitational separation rate and Brownian motion of oil droplets, thereby reducing their collision probability (Boostani et al., 2017). It is also noteworthy that the high viscoelasticity of the interfacial layer could increase the physical stability of the corresponding emulsions (Xiao, Woo, Hu, Xiong, & Zhao, 2021). Accordingly, the higher emulsion stability of black bean protein-sodium alginate conjugates compared to the protein was attributed to the increased mechanical strength and viscoelasticity of the interfacial film (Han et al., 2022).

It is worth noting that the presence of free or non-adsorbed biopolymers (proteins and/ or polysaccharides) in the continuous phase of a protein-polysaccharide conjugate-stabilized emulsion could affect its stability through depletion flocculation. The lowest concentration of biopolymer needed to induce depletion flocculation is called the critical flocculation concentration (CFC), which decreases as a function of the effective volume fraction of the biopolymer and the emulsion droplet size. The flocculation rate firstly increases as a function of free biopolymer concentration due to an increase in the number of the attraction interactions or collisions between the oil droplets; however, once the biopolymer concentration is exceeded a certain concentration, the rate of depletion flocculation is often decreased due to a marked increase in the viscosity of the continuous phase of the emulsion which retards the movement of the oil droplets and collision frequency (McClements, 2015). O’Regan and Mulvihill (2010) studied the storage stability of O/W emulsions stabilized with sodium caseinate (NaCN), maltodextrin (MD)-NaCN mixture, NaCN-MD crude conjugate, and NaCN-MD purified conjugate. The NaCN-stabilized emulsion, with a large increase in the oil droplet size and distribution on storage, was the least stable one (Fig. 2b). The emulsion stabilized with NaCN-MD crude conjugate had the smallest shift in oil droplet size and distribution during storage, and had better stability than the other systems, mainly due to (i) the bulky polymeric layer on the droplet surface that provides better steric stabilization and prevents droplet flocculation and coalescence and (ii) the viscose-enhancing potential of the non-conjugated MD in the continuous phase which decreases the Brownian motion of the droplets and in turn droplet collision frequency. Although the purified conjugate-stabilized emulsion had better storage stability compared to those stabilized by the NaCN and NaCN-MD mixture due to the better steric stabilization function of the interfacial layer, it was less stable than the emulsion stabilized by the crude conjugate which was attributed to the lower concentrations of the non-conjugated MD in the corresponding system; thereby leading to a lower continuous phase viscosity and subsequently higher droplet–droplet interactions (O’Regan & Mulvihill, 2010). Nonetheless, it was reported that although there was an increase in the emulsifying activity of WPI after conjugation with low acyl gellan gum (LAGG) and the WPI-coated droplets had significantly lower storage stability than the conjugate-coated ones, the emulsions stabilized by the WPI-LAGG conjugates (especially 0.3 %LAGG-based ones) were significantly less stable than those stabilized by the WPI-LAGG mixtures (Nooshkam & Varidi, 2020b). This was likely ascribed to (i) the formation of extremely large conjugates with the ability to participate in the interfacial layers of different oil droplets during emulsification which promotes their flocculation *via* bridge formation and/or (ii) the depletion flocculation induced by the non-adsorbed (non-conjugated) polysaccharide molecules in the continuous phase (Hiller and Lorenzen, 2010, Nooshkam and Varidi, 2020b). Therefore, it is necessary to control the reaction parameters to obtain protein-polysaccharide conjugates with the highest emulsion-stabilizing capability to be used as efficient emulsifiers in emulsion-based food products, such as beverage emulsions. As turbidity and phase separation in beverages could substantially affect their consumer acceptance, the use of protein-polysaccharide conjugates could significantly reduce aggregation, sedimentation, and creaming in such products during storage.

### 4.2 pH

Proteins form relatively thin and electrically charged interfacial layers, and electrostatic repulsion is then the main mechanism inhibiting flocculation in protein-coated droplets. Thus, these systems are particularly sensitive to changes in pH, and they tend to flocculate at pH ∼ pI of the adsorbed proteins due to insufficient electrostatic repulsions between oil droplets to overcome the different attractive interactions, such as hydrophobic, Van der Waals, or depletion (Bai et al., 2019, Chen et al., 2016, McClements, 2004). A safe and practical way to upgrade the emulsifying and stabilizing properties of food proteins under both neutral pHs and unfavorable acidic conditions (especially at pHs close to the pI) is by covalent conjugation with hydrophilic polymers such as polysaccharides through the Maillard reaction (Akhtar & Ding, 2017).

In a study, Chen et al. (2016) used wet-heating (WH) and ultrasound-assisted wet heating (UAWH) approaches, in the presence of MD, to improve the emulsifying activity of peanut protein isolate (PPI) under acidic conditions. The O/W emulsions were formed by the untreated PPI, (WH)PPI-MD conjugate, and (UAWH)PPI-MD conjugate at pH 3.8. The PPI-stabilized emulsions had a relatively big droplet size and they were found to be highly flocculated, due to the near-*pI* acidity of the protein, which can result in inadequate electrostatic repulsion, solubility losses, and insoluble protein particles. The (WH)PPI-MD conjugate had limited enhancement in emulsifying properties at acidic pH compared to the untreated PPI. In contrast, the (UAWH)PPI-MD conjugate stabilized emulsions presented the lowest droplet size and they were homogenous with no sign of flocculation. This was attributed to the ability of the ultrasound to increase the accessibility of the functional groups of the major protein subunits and higher levels of PPI can then readily conjugate to MD and remain soluble and surface active at pH ∼ pI. However, the (WH)PPI-MD and (UAWH)PPI-MD conjugates with higher glycation degree formed emulsions with significantly greater droplet size, and this phenomenon was probably ascribed to the strong decrease in surface hydrophobicity at high glycation degree and, in turn, decrease in conjugate’s surface activity (Chen et al., 2016). Therefore, the glycation reaction between polysaccharides and proteins should be carefully controlled to obtain conjugates with improved emulsification and lower contents of advanced glycation products or melanoidins with poor surface activity (Barbosa, Ushikubo, de Figueiredo Furtado, & Cunha, 2019).

The pH tolerance of O/W emulsions stabilized with ASY, BLG-ASY mixture, and BLG-ASY conjugates were investigated at pHs 2, 3, 4.6, and 7, by Bi et al. (2017). For the ASY coated oil droplets, breaking of emulsions was observed at strong acidic pHs (i.e., 2 and 3), indicating that ASY was not able to provide enough emulsion stability against low pHs (Fig. 2c). The oil droplets of emulsions stabilized by BLG-ASY mixture and conjugates with lower heating time (<12 h) experienced strong aggregation at pHs 2 and 3, and this effect was largely attributed to the lower surface charge of the emulsifiers because of the decrease in charged groups at acidic pHs and electrostatic repulsive forces. Moreover, electrostatic interactions between and within the emulsifiers could be changed under acidic conditions, and their adsorption behavior at the O/W interface and emulsion stability can therefore be affected. The emulsions remained homogenous at pHs 4.6 and 7; however, a few large oil droplets were observed at pH 4.6, due to protein charge loss around its *pI*. The BLG-ASY conjugates with high heating time (>12 h) formed emulsions with no particular aggregation at all the pHs studied (Fig. 2c). This could be attributed to the potential of BLG-ASY conjugates to provide enough emulsion stability (e.g., steric and electrostatic repulsion) against acidic pHs (Bi et al., 2017). The improved emulsifying properties of protein-polysaccharide conjugates at acidic conditions have been mainly ascribed to the enhanced steric/electrostatic stabilization and continuous phase viscosity provided by the bulky hydrophilic moiety of the polysaccharide (Akhtar and Dickinson, 2003, Bai et al., 2019, Barbosa et al., 2019). Similarly, Hong, Xiao, Li, Li, and Xie (2022) reported that the average particle size of nanoemulsions stabilized with WPI-dextran conjugates was smaller than nanoemulsions formulated with WPI in the pH range 3–9, suggesting that the conjugates could improve pH stability of nanoemulsions.

In a recent study, the pH stability of O/W nanoemulsions has been evaluated at pHs 7 and 4.6, in the presence of pea protein isolate-dextran and NaCN-dextran conjugates (Caballero & Davidov-Pardo, 2021). Although there were no marked differences between the droplet sizes of protein and conjugate-stabilized emulsions at neutral conditions, the oil droplets coated with conjugates had markedly smaller sizes than the protein-coated counterparts at pH 4.6. Nonetheless, the emulsions stabilized by the conjugates showed significantly lower ζ-potentials at pH 4.6 than the protein stabilized ones; accordingly, it was suggested that the dominant stabilization mechanism switched from electrostatic repulsion in the protein-coated droplets to strong steric hindrance in the conjugate-stabilized pairs (Caballero & Davidov-Pardo, 2021). It is also noteworthy that the lower ζ-potential of the oil droplets coated with conjugates compared to that of the protein-coated counterparts might be ascribed to the fact that dextran is a neutral polysaccharide (Sun et al., 2011). It could therefore have a noticeable effect on the distance from the droplet surfaces (i.e., shear plane) where the effective electrical characteristics are determined. Accordingly, the electrical charge of the dextran-protein conjugate-stabilized oil droplets is measured at a distance that is further from the droplet surfaces and the ζ-potential consequently underwent a greater decay (Gumus et al., 2016, Nooshkam et al., 2022). Another explanation is that the deprotonated carboxylic acid groups (COO^–^) of the polysaccharides can counteract the number of hydrogen ions measured during pH reduction in the conjugate-stabilized emulsions (Zhong et al., 2019).

It has been also reported that although the WPI-LAGG conjugates formed O/W emulsions with generally higher stability against pH variations compared to the native WPI, the droplet size of the emulsions containing conjugate at pH 3 was remarkably greater than those stabilized by the native protein. This was explained probably by the formation of irreversible droplet flocculation and gel-like structures in the system as a result of strong electrostatic interactions between negatively charged LAGG and positively charged protein molecules, when the emulsions had to pass through the *pI* of the protein (Nooshkam & Varidi, 2021a). In general, the magnitude and range of the steric repulsion between oil droplets of the emulsions are mainly determined by the thickness of emulsifier molecules on the surface of droplets; the thicker the interfacial layer, the stronger and longer-range the steric repulsion. The superb emulsion stability of protein-polysaccharide conjugate-based emulsions at low pHs (especially at pH ∼ pI) could be, therefore, mainly ascribed to the strong steric repulsion, which could countervail the prevailing Van der Waals attraction between oil droplets, thereby inhibiting flocculation and phase separation (Zha et al., 2019b, Zhong et al., 2019). The protein-polysaccharide conjugates could be therefore employed as outstanding emulsifiers to inhibit droplet aggregation in emulsion-based food products under acidic conditions and maintain their homogeneity throughout the shelf-life. This could mainly be due to the fact that the Maillard reaction is able to switch the dominant stabilization mechanism from electrostatic repulsion in the protein-coated droplets to strong steric hindrance in the conjugate-stabilized pairs.

### 4.3 Salt stability

Emulsions can be incorporated into food products containing different ionic compositions, and the stability of such emulsions to high ionic strengths should be therefore evaluated (Liu et al., 2016). Biopolymer-coated oil droplets are mainly stabilized by electrostatic repulsion, and the surface charge of the droplets may be reduced by the addition of counter-ions and electrostatic screening phenomenon. At this point, the attractive forces such as hydrophobic and Van der Waals, may dominate the repulsive forces and in turn result in droplet aggregation (Wei & Gao, 2016). Many types of proteins could be applied as emulsifiers owing to their amphiphilic properties, polymeric structure, and electrical charge characteristics. Nonetheless, the emulsions stabilized by proteins are greatly susceptible to environmental stresses like ionic strength and they, therefore, undergo flocculation destabilization at high ionic strength, limiting their usage in some commercial products (Burgos-Díaz, Wandersleben, Marqués, & Rubilar, 2016). The research has shown that emulsions stabilized by the Maillard-based protein-polysaccharide conjugates present high resistance to ionic strength (Barbosa et al., 2019). The polysaccharide moiety of the protein-polysaccharide conjugate can provide sufficient additional steric stabilization to overcome any attractive interactions between oil droplets induced by unfavorable conditions of ionic strength (Dickinson, 2008, Han et al., 2019).

In a study conducted by Zhang, Guo, Zhu, Peng, and Zhou (2015), although droplet size increased as a function of NaCl concentration, the O/W emulsion stabilized by oat protein isolate-dextran conjugate was more stable to high ionic strength (200 mM) compared to those containing heated protein and native protein. This increase in droplet size is well correlated to the reason that the high ionic strength-induced electrostatic screening results in lower electrostatic repulsions between oil droplets; however, the better salt stability of conjugate-coated oil droplets is mainly due to the fact that the protein-polysaccharide conjugate is able to enhance the steric repulsive forces and decrease the Van der Waals attractions between the droplets (Zhang et al., 2015). Consistently, the emulsion stabilized with arabinoxylan hydrolysate-SPI conjugate was found to show the best salinity tolerance because the conjugate can inhibit the electrostatic shielding of salt ions and prevent the reduction of the electrostatic repulsion force between emulsion droplets. The conjugate can form a multilayer interface to prevent oil migration from droplet to droplet, thereby inhibiting droplet aggregation (Li et al., 2022).

It is noteworthy that counter-ions (Na^+^) may accumulate loosely around the carboxylic groups (–COO^-^) on the protein surfaces and screen their net charge. The lower change in ζ-potential of protein-polysaccharide conjugate stabilized colloidal suspensions as a function of ionic strength maybe therefore attributed to the presence of polysaccharide bulky chains on the droplet surface that highly decreases the movement of the droplets upon subjecting to the electric field (Du et al., 2013). Moreover, it has been reported that the hydration layer of the outer of proteins can be induced to be thin at high salt ion concentrations, leading to protein molecules’ agglomeration and in turn increase in droplet size of emulsions (Bai et al., 2019). The presence of protein-polysaccharide conjugate on the oil droplet surface could reduce the electrostatic shielding effect of salt ions. In this context, the salt-stability of emulsions prepared with pea protein isolate, pea protein isolate-gum Arabic mixture, and pea protein isolate-gum Arabic conjugate was investigated under different ionic strengths (0, 100, 300, and 500 mM NaCl) (Zha, Dong, Rao, & Chen, 2019a). The protein stabilized emulsion was generally less stable at >100 mM ionic strength, with a cream layer. The main reason is that the charge around oil droplets is likely shielded by salt ions at high concentrations. The emulsion stabilized by protein-polysaccharide mixture showed a similar trend, but with a lesser extent and no phase separation. Nonetheless, the emulsions containing conjugates were still homogeneous in the presence of 500 mM salt. The conjugated protein-polysaccharide is therefore able to improve the salinity-tolerance of the emulsion effectively, due most probably to the improved electrostatic and steric repulsive forces (Zha et al., 2019a). Similarly, it has been demonstrated the WPI-LAGG conjugate stabilized emulsions were more homogenous with markedly smaller droplet size change than those stabilized with WPI and WPI-LAGG mixture, and this effect was ascribed to the formation of thicker interfacial layers and improved steric repulsion between the oil droplets (Nooshkam & Varidi, 2021a).

The hairy carbohydrate protrusions on the surface of protein-polysaccharide conjugate-coated droplets in colloidal suspensions could generally provide a strong steric effect and hamper the formation of salt bridges and finally particle aggregation under high ionic strengths (Han et al., 2019). The high salinity tolerance of protein-polysaccharide conjugate-stabilized emulsions strongly implies that the corresponding systems are stabilized by different forces, particularly steric repulsion, which arise from the various properties of the interfacial layer surrounding the oil droplets. The stable and homogenous emulsions prepared with protein-polysaccharide conjugates are generally resistant to electrolytes and could be used in high ionic strength food systems. However, it should be taken into account that the fortification of foods and beverages with minerals such as calcium or iron is a common practice in the food industry. At this point, gel structures based on reversible and/or irreversible bridging flocculation with divalent cations could occur in the emulsions containing proteins and polysaccharides, particularly anionic polysaccharides. Nonetheless, the conjugate-stabilized emulsions are generally more resistant to droplet flocculation and aggregation in the presence of both mono- and divalent cations in comparison to the protein-stabilized counterparts (Caballero & Davidov-Pardo, 2021).

### 4.4 Thermal stability

Food and beverage products often experience a range of temperatures during production, transport, storage, and utilization (Gumus et al., 2016). Emulsions, especially protein-stabilized ones, are highly sensitive to thermal treatment (Bi et al., 2017). The thermal stability of protein-based emulsifiers is of growing concern because a positive correlation has been found between thermal stability and techno-functional properties of this type of emulsifiers, such as solubility, emulsifying capacity, foaming, and creaming stability (A'Yun et al., 2020). Proteins denature upon heating above denaturation temperature and subsequently undergo aggregation, which leads to viscosity increment in protein-stabilized O/W emulsions. The interfacial protein is also denatured at high temperatures, thereby increasing attractive interactions between oil droplets (A'Yun et al., 2020, Bi et al., 2017). The denaturation of globular proteins leads to structural changes and exposure of thiol and hydrophobic groups, which may induce protein–protein interactions and aggregation of protein-coated oil globules in emulsions. Nevertheless, such aggregations could be physically restricted by the presence of a conjugated form of the protein at the O/W interface of oil droplets due to steric stabilization (O’Mahony et al., 2019). The protein-polysaccharide conjugates are amphoteric hybrids with the potential to achieve surface layer saturation at a much lower bulk concentration and the adsorbed protein layer is protected against destabilization under unfavorable conditions, such as heating (Liu, Zhao, Zhao, Ren, & Yang, 2012).

Accordingly, it has been reported that SPI-dextran conjugate was able to markedly increase the stability of emulsions against heat-induced aggregation in boiling water. And this effect was attributed to the ability of dextran molecules located at the oil droplet surfaces to prevent the interaction between droplet surface membrane and aggregated protein in the continuous phase. Moreover, it is expected that the protein tends to resist denaturation and aggregation upon conjugation with the polysaccharide (Diftis & Kiosseoglou, 2006). Indeed, the Maillard reaction can lower the sensitivity of food proteins to heating treatments by inhibiting intermolecular interactions between protein molecules and preserving the structure of the native protein. Additionally, the polysaccharide part of the protein-polysaccharide conjugate could lower the solvent availability of proteins through the crowding effect and force the protein molecules to adopt a compact and steady-state structure. Furthermore, the conjugated proteins could be highly stable to thermal treatments in the presence of anionic polysaccharides, inducing steric and electrostatic repulsive forces and, in turn, inhibiting protein aggregation during the conjugation reaction (Nooshkam et al., 2020). In this context, the Maillard reaction-based conjugates have been successfully used to inhibit the droplet size change and aggregation of milk protein-stabilized emulsions under high temperatures (A'Yun et al., 2020, Al-Hakkak and Kavale, 2002).

Bi et al. (2017) demonstrated that no changes in particle size distribution and bulk phase separation were observed for O/W emulsions stabilized by BLG-ASY conjugates with high incubation time (>12 h) under high temperature and salt concentrations, in comparison to control (BLG-ASY-0 h) and the conjugates of <12 h incubation time, which experienced phase separation under conditions applied (Bi et al., 2017). In our previous work, it was found that the O/W emulsions stabilized by WPI, WPI-LAGG mixture, and WPI-LAGG conjugates generally experienced a marked rise in oil droplet size (up to 35.33%) and PDI (up to 84.77%) with increasing heating temperature from 50 to 90 °C; however, the emulsion stabilized by WPI showed significantly higher droplet size and PDI changes after thermal treatment compared to other systems, particularly above the denaturation temperature of the proteins molecules (≥70 °C) (Nooshkam & Varidi, 2021a). This effect could be mainly assigned to the denaturation of globular proteins and in turn, the exposure of hydrophobic and free sulfhydryl groups, which have the potential to induce droplet aggregation through disulfide bonds and hydrophobic interactions between protein molecules on the droplet surfaces (McClements, 2015). The conjugate-stabilized emulsion had, however, a significantly lower alteration in droplet size (6.67%) than those containing WPI (25.15%) and mixture (20.26%) (Nooshkam & Varidi, 2021a), mainly due to (1) the thermal stability of the conjugated protein and (2) improved steric repulsive forces between oil droplets as a consequence of thicker and cohesive membrane films around oil droplets (Nooshkam and Varidi, 2021a, Zha et al., 2019a). A similar trend was reported by Chen et al. (2022), who showed that WPI-gum Acacia conjugate-stabilized emulsions had better thermal stability (in boiling water for 30 min) compared to WPI- and WPI-gum Acacia mixture-stabilized emulsions.

**Fig. 3:**
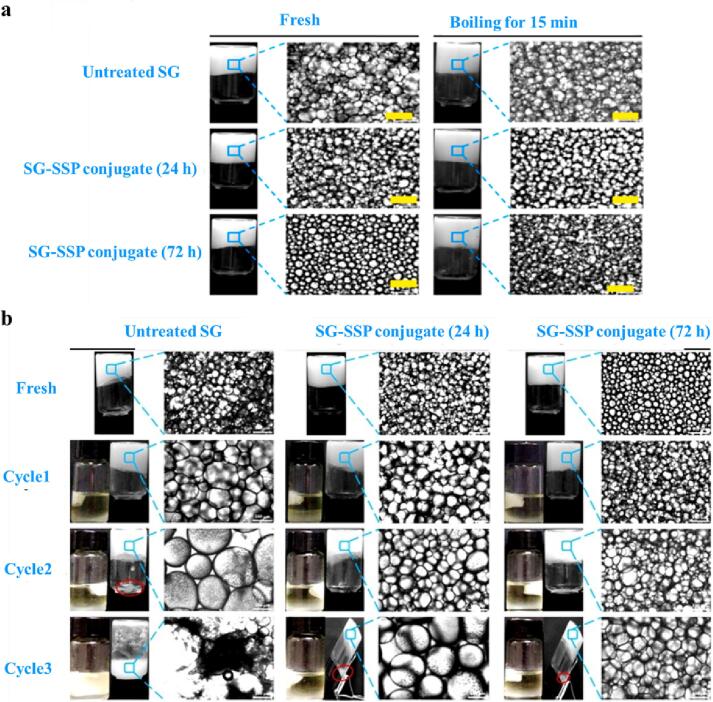
Visual observation and optical micrographs of the high internal phase emulsions stabilized by untreated soy glycinin (SG), or SG glycated with soy soluble polysaccharide (SSP) for 24 and 72 h, (a) without or with heating in boiling water for 15 min and (b) before or after different cycles of freeze–thaw treatment. Reproduced from ref. (Hao et al., 2020) with permission from Elsevier Science Ltd. Copyright 2020.

In a recent work conducted by Hao, Peng, and Tang (2020), it has been reported that the conjugation of soy glycinin (SG) with soy soluble polysaccharide (SSP) provided SG-SSP conjugates with improved emulsifying properties and gel network stability (against heating) in high internal phase emulsions (HIPEs). The HIPEs stabilized by the SG-SSP conjugate were highly heat-stable (Fig. 3a). The increased emulsification properties of the SG by the conjugation reaction were greatly attributed to the enhanced adsorption and the dissociation of the protein subunits at the interface (Hao et al., 2020). Similarly, Tirgarian, Farmani, Farahmandfar, Milani, and Van Bockstaele (2022) reported that HIPEs stabilized by SPI or NaCN and *Alyssum homolocarpum* seed gum or kappa-carrageenan conjugates were ultra-stable against thermal treatment (90 °C for 30 min). The improved heat stability of food emulsions stabilized by proteins as a result of conjugation reaction has been also reported in other studies (A'Yun et al., 2020, Drapala et al., 2016, Zhang et al., 2023, Zhang et al., 2022). It can be concluded that the ability of protein-polysaccharide conjugates to form a cohesive, impermeable, thick, and heat-stable interfacial layer around oil droplets, makes them ideal functional emulsifiers for heat-treated emulsion-based food products.

### 4.5 Freeze-thaw treatment

Many types of O/W emulsion foods are necessarily subjected to frozen treatments during their production process to delay biochemical processes, inhibit undesirable microbial activity, and increase the shelf life of final products (Wang, Zhang, Jiang, Zhao, & Fu, 2020). Most the emulsions prepared with native proteins are physically unstable (Zhang et al., 2017). Protein-stabilized emulsions undergo a series of physicochemical changes during freeze–thaw treatments, mainly due to ice crystal formation. At this point, the interface film may be destroyed by the sharp ice crystals, and at the same time, the released interfacial protein could adsorb onto the ice crystals, leading to insufficient protein adsorption. Subsequently, several phenomena such as flocculation, coalescence, unstable oil release, and even complete phase separation could occur during emulsion thawing (Wang, Zhang, Wang, Xu, & Jiang, 2020). In addition, low temperatures could result in protein denaturation, and their functionality may be subsequently lost during emulsion thawing (Nooshkam & Varidi, 2021a). A thick interfacial layer could provide better stability for protein-stabilized emulsions. In this context, the attachment of a steric polysaccharide layer to the protein through the Maillard reaction could generally provide a relatively thick interfacial layer on the droplet’s surface of protein-stabilized emulsions, with the potential to resist disruption and inhibit droplets from coming into proximity (Xu et al., 2010).

The casein hydrolysates-carboxymethyl chitosan conjugate stabilized O/W nanoemulsions, despite a partial rise in the particle size, were found to be more homogeneous with no bimodal distribution after several freeze-thawing treatments; however, casein-stabilized ones experienced a significant increase in particle size and bimodal size distribution (0.1–1 μm) after multiple freeze–thaw cycles. And this has been attributed to the formation of a viscoelastic layer at the interface by the conjugate (Wang et al., 2020). Novel ternary conjugate-based emulsifiers were designed by Liu et al. (2016), through covalently bonding chlorogenic acid (CA), lactoferrin (LF), and dextran together. The LF and CA-LF conjugate stabilized emulsions experienced large-scale coalescence and gravitational separation after the freeze–thaw treatment. However, the emulsions containing LF-Dextran or CA-LF-Dextran conjugates were highly stable to freeze–thaw treatment and seemed to be more homogeneous, mainly due to the steric repulsive forces between oil droplets provided by the polysaccharide dextran (Liu et al., 2016). Similarly, Xu, Wang, Jiang, Yuan, and Gao (2012) reported that the freeze–thaw stability of WPI-stabilized O/W emulsions could be significantly improved when the WPI-beet pectin Maillard reaction-based conjugates were used as emulsifiers. This was ascribed to the hydrodynamic and steric stabilization effects provided by the thicker interfacial membranes, inhibiting partial coalescence under unfavorable conditions (Xu et al., 2012). Polysaccharide moiety of protein-polysaccharide Maillard conjugates have also been reported to increase unfrozen water content in continuous phases of emulsions, preventing droplets from being forced into close proximity (Chen et al., 2022). Soy protein hydrolysates-dextran conjugates showed a similar effect on the stability of food emulsions against freeze–thaw treatments (Zhang, Yu, Wang, Wang, & Zhang, 2019). In an interesting study, it was found that the conjugation markedly improved the reversibility of destabilization (upon freeze-thawing) and re-emulsification of the protein-based HIPE gel, by enhancing the structural stability of the protein (or its subunits) (Fig. 3b) (Hao et al., 2020). The Maillard reaction-based protein-polysaccharide conjugates could therefore form thick interfacial films on the oil droplets and prevent instability of food emulsions during the multiple freeze–thaw cycles. Due to its shielding/covering effect, the polysaccharide moiety in the conjugate could also increase the protein's resistance to cold denaturation.

### 4.6 Dilution

Beverage and other liquid foods are usually subjected to dilution treatments, and the storage and transportation of such products are influenced accordingly. In this regard, beverage materials with good dilution behaviors could guarantee product quality and save production costs because they could be stored and transported in a concentrated form. The droplets of concentrated emulsions could be disrupted upon dilution. Moreover, the dilution treatment could affect nutrient stability (Ding et al., 2020). Thus, it is mandatory to design food emulsions with high stability to dilution treatments. The protein-polysaccharide conjugates could be considered superb candidates for this purpose. On this point, the impact of emulsifier type on the dilution stability of food emulsions has been investigated by Xu, Yuan, Gao, McClements, and Decker (2013). The continuous phase of O/W emulsions stabilized by WPI-beet pectin Maillard reaction-based conjugate and unconjugated mixture (non-heated WPI-beet pectin mixture) were washed three times with phosphate buffer (10 mM, pH 7) and the droplet size distribution of the emulsions was monitored. Unlike the conjugate-stabilized emulsion, the unconjugated mixture-based one experienced a remarkable rise in the droplet size after the washing process, mainly due to the increased interactions between droplets and subsequent coalescence. The high stability of the washed conjugate emulsion to dilution might be ascribed to the thick and cohesive interfacial layer of the corresponding system. Moreover, β-carotene was preserved more effectively in the washed conjugate emulsion than in the mixture-stabilized one. Indeed, the conjugate-based interfacial layer may function as a physical barrier between prooxidants (such as iron) in the continuous phase and oxidizable β-carotene within the droplets’ core (Xu et al., 2013). Therefore, the protein-polysaccharide conjugates could be used in the food industry to design highly stable beverages and emulsions, particularly beverage emulsions, to dilution treatments and nutraceutical degradation. It would be possible to dilute and/or incorporate these protein-polysaccharide conjugate stabilized beverage emulsions as water-insoluble ingredient carriers in beverage and drink formulations.

### 4.7 Chemical stability

Fat and oil often exist as emulsions in processed foods. Lipid oxidation has a remarkable effect on the stability of emulsions in food, and it is therefore considered one of the important issues in food storage and consumption, with the potential to influence the flavor, odor, and color of food (Nooshkam et al., 2019, Ries et al., 2010). Oils have to be shielded to inhibit them from going stale due to their susceptibility to oxidation, which develops off-flavors and odors, and in turn nutritional value losses (Li, Woo, Patel, & Selomulya, 2017). The emulsion stability and interfacial layer thickness of the droplets are contributed to the antioxidant behavior in O/W emulsions. Accordingly, the thicker interfacial layer could act as a physical barrier between free radicals and oil droplets (Shi et al., 2019). The Maillard reaction-born protein-polysaccharide conjugates could be recommended as outstanding emulsifiers and interfacial antioxidants in food products containing O/W emulsions to inhibit the oil oxidation or the degradation of bioactive compounds.

In this context, ovalbumin (OVA) and OVA-inulin conjugates were employed to stabilize pomegranate seed oil emulsions. The glycated OVA emulsion system had significantly higher oxidation stability than the OVA emulsion, due to the ability of the glycated protein to form a dense, thick, and viscoelastic physical barrier (i.e., interfacial protective film), hindering free radical migration to the droplet cores and slowing down the lipid oxidation rate (Hu et al., 2020). It was also found that emulsion droplets coated with WPI-polysaccharide conjugate exhibited better oxidative stability than WPI with less peroxide produced after accelerated oxidation for 7 days (Yu-Tong, Chun, Yue-Ming, Bao, & Xiong, 2022). Moreover, it has been reported that LF, LF-CA binary conjugate, and LF-CA-Dextran ternary conjugate show different inhibitory rates against β-carotene degradation in O/W emulsions. β-Carotene degradation followed the order: LF > binary conjugate > ternary conjugate-coated oil droplets. The highest β-carotene protection in the ternary conjugate-coated oil droplets of O/W emulsions has been attributed to (i) the presence of functional groups in the conjugate that function as radical-scavenging agents, (ii) the ability of the protein moiety of the conjugate (e.g., lactoferrin) to chelate pro-oxidant transition metals, and (iii) the potential of the ternary conjugate in the formation of thick and dense interfacial films on the droplet surfaces, inhibiting pro-oxidants from interacting with the lipids physically (Liu et al., 2016). It could be also noteworthy that the protein-polysaccharide conjugates can successfully encapsulate bioactive materials such as fish oil, conjugated linoleic acid, and nutraceuticals (Chen et al., 2022, Huang et al., 2020, Jia et al., 2020, Kan et al., 2022, Li et al., 2017, Nooshkam and Varidi, 2021a). In this regard, the conjugates could act as antioxidants and increase the wall integrity of the microcapsules, retarding the penetration of oxygen into the microcapsules and improving the chemical and storage stability of the bioactive compound.

## 5 Microbial stability of protein-polysaccharide conjugate-stabilized emulsions

It is also important to consider the microbial stability of emulsion-based foods along with their physical and chemical stability. The use of synthetic antimicrobials has been extensively studied for extending the shelf life of emulsion-based foods. However, their possible carcinogenic, allergic, and genotoxic effects have led to growing concerns about their safety. Hence, the food scientists are discovering and developing novel antimicrobial compounds that are natural and low-cost in response to customer demand for natural ingredients (Kumar et al., 2016, Verma et al., 2018, Wang et al., 2016). It has been shown that Maillard conjugates, particularly chitosan-based derivatives, could increase the shelf-life and microbiological safety of food products, such as meats, fresh shrimps, and noodle (Nooshkam et al., 2020). There is, however, little knowledge of the antimicrobial activity of protein-polysaccharide Maillard conjugates in emulsified foods. In recent years, more research has been conducted on conjugating lysozyme to polysaccharides to improve its antimicrobial properties against both Gram-positive and Gram-negative bacterial species (Aminlari et al., 2005, Hamdani et al., 2018, Hashemi et al., 2014). In a study, lysozyme was conjugated with gum Arabic to be used in mayonnaise, which requires both preservatives and emulsifiers. In addition to improved functional properties, the lysozyme-gum Arabic conjugate also demonstrated antibacterial activity. Applied to mayonnaise, the lysozyme-gum Arabic conjugate increased the death rate of *Staphylococcus aureus* and *Escherichia coli K*-12. The increased emulsification role of lysozyme-gum Arabic conjugate with a higher elasticity in mayonnaise was confirmed by investigations of emulsion stability and rheological properties. Moreover, the rheological response of mayonnaise containing conjugates was linear, and the product showed shear-thinning behavior. Interestingly, the sensory analysis of the lysozyme-gum Arabic conjugated mayonnaise was in complete agreement with that of the commercial product (Hashemi et al., 2018). The antimicrobial effect of Maillard conjugates has been mainly ascribed to their metal chelation and surface-active properties (Nooshkam et al., 2019, Nooshkam et al., 2020). A protein-polysaccharide conjugate interface framework could therefore confer high physical, chemical, and microbial stability to food emulsions.

## 6 Conclusion and future trends

Protein-polysaccharide conjugates can locate at O/W emulsion’s interfaces, where they could stabilize emulsions against destabilization factors through different physicochemical mechanisms, including steric and electrostatic repulsive forces. Although protein-polysaccharide conjugates designate a promising class of emulsifiers with potential application in the food industry, some challenges remain. The composition and structure of protein-polysaccharide conjugates should be first well known to control the underlying reactions. On this point, advanced analytical methods should be applied to identify the exact composition and structure of protein-polysaccharide conjugates, and their location in the food emulsions. Thus, the interfacial properties of the conjugates could be predicted easily. Moreover, the impact of certain protein-polysaccharide conjugates, especially those obtained in the advanced stage of the Maillard reaction, on human health remains a concern. Therefore, it is essential to control the Maillard reaction to obtain protein-polysaccharide conjugates with a minimum level of harmful compounds and the highest emulsifying properties. The feasibility of protein-polysaccharide conjugates in industrial applications requires more studies. In this context, sustainable preparation processes and the economic profitability of the conjugates should be carefully designed and evaluated, respectively. In general, the Maillard reaction-based protein-polysaccharide conjugates could be used to design functional foods with improved physical, oxidative, and microbial stability.

## Declaration of Competing Interest

The authors declare that they have no known competing financial interests or personal relationships that could have appeared to influence the work reported in this paper.

## Data Availability

No data was used for the research described in the article.
